# Performance of Sokolow-Lyon index in detection of echocardiographically diagnosed left ventricular hypertrophy in a normal Eastern German population - results of the CARLA study

**DOI:** 10.1186/s12872-015-0066-5

**Published:** 2015-07-14

**Authors:** Jochen Schröder, Sebastian Nuding, Ursula Müller-Werdan, Karl Werdan, Alexander Kluttig, Martin Russ, Karin H. Greiser, Jan A. Kors, Johannes Haerting, Daniel Medenwald

**Affiliations:** Department of Medicine III, Martin-Luther-University Halle-Wittenberg, University Hospital Halle / Saale, Ernst-Grube-Str. 40, 06120 Halle/Saale, Germany; Martin-Luther-University Halle-Wittenberg, Institute of Medical Epidemiology, Biostatistics and Informatics, Magdeburger Str. 8, 06112 Halle/Saale, Germany; Department of Cardiology and Pneumology, Helios Amper Kliniken Dachau, Krankenhausstr.15, 85221 Dachau, Germany; Division of Cancer Epidemiology, German Cancer Research Centre, Im Neuenheimer Feld 581, 69129 Heidelberg, Germany; Department of Medical Informatics, Erasmus Medical Center Rotterdam, Kamer Na-2617, Postbus 2040, 3000 CA Rotterdam, The Netherlands; Clinic for Geriatric Medicine of the University Hospital RWTH Aachen, Aachen, Germany

**Keywords:** Left ventricular hypertrophy, Hypertrophy, ECG, Sokolow-Lyon index, Sokolow, Obesity

## Abstract

**Background:**

Arterial hypertension is a common disease with high prevalence in the general population. Left ventricular hypertrophy (LVH) is an independent risk factor in arterial hypertension. Electrocardiographic indices like the Sokolow-Lyon index (SLI) are recommended as diagnostic screening methods for LVH.

We assessed the diagnostic performance of the SLI in a cohort of a large general population.

**Methods:**

We used electrocardiographic and echocardiographic data from the prospective, population-based cohort study CARdio-vascular Disease, Living and Ageing in Halle (CARLA). Linear and logistic regression models were used to assess the association of SLI with LVH. To assess the impact of the body-mass-index (BMI), we performed interaction analyses.

**Results:**

AUC of SLI to predict LVH was 55.3 %, sensitivity of the SLI was 5 %, specificity 97 %. We found a significant association of SLI after covariate-adjustment with echocardiographically detected LVH (increase of left-ventricular mass index, LVMI 7.0 g/m^2^ per 1 mV increase of SLI, *p* < 0.0001). However, this association was mainly caused by an association of SLI with the left-ventricular internal diameter (LVIDd, increase of 0.06 cm/m^2^ per 1 mV increase of SLI, *p* < 0.0001). In obese (BMI > 30 kg/m^2^) we found the strongest association with an increase of 9.2 g/m^2^ per 1 mV.

**Conclusions:**

Although statistically significant, relations of SLI and echocardiographic parameters of LVH were weak and mainly driven by the increase in LVIDd, implicating a more eccentric type of LVH in the collective. The relations were strongest when obese subjects were taken into account. Our data do not favour the SLI as a diagnostic screening test to identify patients at risk for LVH, especially in non-obese subjects without eccentric LVH.

## Background

Arterial hypertension is a common disease with a prevalence of 30–45 % in the general population in Europe [1]. A recent publication reported a prevalence of 44.2 % in the general population in Europe with the highest prevalence of 55.3 % in Germany [2]. There is increasing evidence of regional differences with a higher prevalence of arterial hypertension in the eastern part of Germany [3].

In patients with arterial hypertension, LVH initially serves as a compensatory mechanism of the heart to deal with the increased arterial pressure. In recent guidelines LVH is defined as an increase in left ventricular mass (LVM) > 115 g/m^2^ in men and > 95 g/m^2^ in women [1].

Increase of LVM is an independent risk factor in arterial hypertension. In patients with arterial hypertension without pre-existing cardiac disease and myocardial wall thickening, the 10-year mortality rate rises up from 1 to 6 %, with an increase in LVM of 10 % up to 24 % in case of a concentric or eccentric hypertrophy [4]. Thus, for patients suffering from arterial hypertension the diagnosis of a possibly existing LVH is of essential importance.

From the early beginnings of LVH diagnosis by means of Chest X-ray [5], nowadays, (transthoracic) echocardiography or magnetic resonance imaging (MRI) became gold standards to assess left ventricular size and mass [1]. Based on myocardial wall thickness, LVM can be estimated easily, however, echocardiography usually has to be performed by a trained cardiologist. A transfer of the patient to a specialist is therefore usually mandatory, which is associated with increased costs and waiting times for the patient. Consequently, these circumstances weaken its value as a practical screening examination in the general population.

For a long time, the 12-lead-electrocardiogram (ECG) is considered a cost- and time-effective alternative in comparison to echocardiography or MRI for detecting LVH by using various indices. Traditionally, the 10 s-ECG is recommended to screen patients with arterial hypertension for LVH [1]. The most popular index was developed and published in 1949 by Maurice Sokolow and Thomas P. Lyon, and is calculated as the sum of the amplitudes of the S-wave in V_1_ and the R-wave in V_5_ or V_6_ (whichever is larger) [6]. In current (non-cardiologic, general practitioner care) routine, the Sokolow-Lyon index (SLI) by a threshold of 3.5 mV is widely used as a measure for LVH.

All of the hypertrophy indices, in particular the SLI, show high specificity but low sensitivity to varying degrees, which weakens its diagnostic value [5]. However, most of the data reported were derived from arterial hypertension patient collectives, while reliable data from population-based cohort studies are still missing.

The aim of this study was to investigate the diagnostic performance of the Sokolow-Lyon index for diagnosis of LVH in a large cohort of the general population.

## Methods

### Study population

We used data from the *CARdio-vascular Disease, Living and Ageing in Halle* (CARLA) study, which is a prospective population-based cohort study of the general population of the city of Halle in Saxony-Anhalt in the Eastern part of Germany [7, 8]. The study was approved by the Ethics Committee of the medical faculty of the Martin-Luther-University Halle-Wittenberg and by the State Data Privacy Commissioner of Sachsen-Anhalt. The CARLA cohort comprises 1779 participants aged 45–83 years at baseline (812 women, 967 men). In order to focus on the middle-aged and elderly population subjects older than 45 years were considered for the study. A multi-step recruitment strategy aimed to achieve a high response rate. The final response rate percentage after subtracting exclusions (individuals who were deceased prior to the invitation, had moved away, or were unable to participate due to illness) was 64 %. All data used in this cross sectional analysis resulted from the baseline examination of the study. The baseline examination took place between December 2002 and January 2006. The study participants underwent a detailed medical examination and a standardized, computer-assisted interview, which collected information on socio-demographic and socioeconomic variables, behavioral, biomedical, and psychosocial factors, medical history, and the use of medication within the preceding 7 days. Furthermore, anthropometric, hemodynamic (blood pressure, ankle-brachial-index and heart rate), electrocardiographic and echocardiographic data were obtained in every participant. A more comprehensive account of the CARLA study can be found elsewhere [7].

### ECG recording and processing

12-lead ECGs were recorded for 10 s after a supine resting period of at least 20 min. All ECGs were processed by the Modular ECG Analysis System (MEANS) to obtain locations and amplitudes of the QRS-complexes required for the computation of the SLI [9]. The good performance of the MEANS has been demonstrated in several studies [10, 11].

### Echocardiographic assessment

Transthoracic Doppler echocardiographic examinations were conducted and evaluated by specially trained and certified physicians. All echocardiographers underwent the same dedicated study certification procedures. The mean intra-observer bias for the M-mode examinations varied between 0.3 and 3.8 % (2*SD between 15.3 and 27.7 %), while the inter-observer variability ranged between 0.1 and 2.7 % (2*SD between 12.7 and 20.8 %). All echocardiographic examinations at baseline were performed using a GE ultrasound system (General Electric Company, Fairfield, CO, USA, GE Vivid 4 and 5). To calculate the LVM, we used the formula of the American Society of Echocardiography (ASE-cube formula), which is in accordance with international guidelines [1, 12, 13]. We also assessed the component variables of the formula, which in the clinical routine often serve as surrogate for LVH: septal wall thickness at end-diastole (SWTd), left ventricular posterior wall thickness at end-diastole (PWTd) and the left-ventricular internal dimension at end-diastole (LVIDd). The measurement of all LVM compound variables was done in the m-mode of the parasternal long axis. The precision used for calculation was 0.1 mm.

### Statistical methods

Results in Table 1 are given as geometric means with their respective 95 % confidence interval (95 % CI). We used linear regression models to assess the association of echocardiographic components with SLI. Categorizing the SLI (cut off: 3.5 mV), we applied logistic regression models to compute odds ratios (OR) for its association with echocardiographic parameters of LVH (mildly impaired or above [13]). Models were adjusted for potential confounders which were history of myocardial infarction, presence of diabetes mellitus, NT pro-BNP, low density lipoprotein, regular intake of beta-blockers, digitalis glycosides, and anti-arrhythmic drugs (ATC code: C01B), presence of arterial hypertension (blood pressure > 140/90 mmHg, or use of anti-hypertensive medication), waist-to-hip ratio and sport index. [14] Confounders were selected using directed acyclic graphs (DAGs) as it is recommended for epidemiological studies [15].

**Table 1:** Basic characteristics

	Men			Women		
Parameter	n	mean	(95 % CI)	n	mean	(95 % CI)
SLI (mV)	950	2.1	(2.1–2.1)	808	2.2	(2.2–2.3)
age (years)	967	64.0	(63.4–64.7)	812	63.0	(62.3–63.7)
QTc (ms)	950	425.0	(423.6–426.6)	808	427.8	(426.2–429.4)
LVM (g)	909	120.03	(118.1–122.1)	784	105.3	(103.5–107.1)
LVMI (g/m^2^)	909	236.6	(230.4–240.9)	784	185.0	(181.4–188.6)
BMI (kg/m^2^)	867	27.9	(27.6–28.1)	812	28.1	(27.7–28.4)
SBP (mmHg)	966	144.5	(143.3–145.8)	812	140.0	(138.5–141.5)
DBP (mmHg)	966	85.2	(84.5–85.9)	812	82.5	(81.8–83.3)
Prevalence data						
	n	%		n	%	
SLI	41	4.6 %		31	4.0 %	
LVH	675	81.2 %		622	83.2 %	
Hypertension	682	76.4 %		555	71.3 %	
BMI						
BMI I	202	22.6 %		212	27.3 %	
BMI II	435	48.7 %		298	38.3 %	
BMI III	256	28.7 %		268	34.5 %	

*SLI* Sokolow-Lyon index, *LVM* left-ventricular mass, *LVMI* left-ventricular mass index; *BMI* body-mass-index, *SBP* systolic blood pressure, *DBP* diastolic blood pressure, *QTc* corrected QT-interval, 95 % *CI* 95 % confidence interval, *LVH* left ventricular hypertrophy, *BMI I* BMI: <25 kg/m^2^ (normal), *BMI II* BMI: 25–30 kg/m^2^ (overweight), *BMI III* BMI: >30 kg/m^2^ (obese) [16]

Furthermore, areas under the receiver operation characteristics curves (AUCs) were computed. Additionally, we tested for interactions of LVMI and BMI (according to the recommendations of the World Health Organization, BMI was categorized into the classes of < 25 kg/m^2^ (class I), 25–30 kg/m^2^ (class II), > 30 kg/m^2^ (class III) [16]) on a multiplicative scale by including the corresponding interaction terms in the respective regression models. To respect physiological relations, presented p-values of the SLI/BMI interaction in Table 3 refer to BMI considered on a continuous scale rather than categories (effect estimates presented in the main text if statistically significant). We assessed violations of the linearity assumption using restricted cubic splines, which indicated that linearity was an adequate fit to the data.

LVH was defined as a left ventricular mass index (LVMI, LVM divided by the body surface area) above 115 g/m^2^ in men and above 95 g/m^2^ in women [1].

We performed a sensitivity analysis where we adjusted for age and sex only in order to assess the confounding effect of these two parameters (results presented in the appendix).

All statistical analyses and data management were performed using SAS®, Version 9.3 (SAS Inc., Cary, NC, USA).

## Results

Data of the 1779 participants of the baseline investigation were included in the statistical analysis. Baseline data of the CARLA study, including prevalences of cardiovascular risk factors like arterial hypertension, are published elsewhere [8, 17].

Mean age was 63.6 years (95 % CI: 63.1–64.0). The mean SLI was 2.2 mV (95 % CI: 2.1–2.2). In the whole study collective, the mean systolic blood pressure (SBP) was 142.3 mmHg (95 % CI: 141.5–143.4), while mean diastolic blood pressure (DBP) was 84.0 mmHg (95 % CI: 83.4–84.5). In men, mean SBP was 144.5 mmHg (95 % CI: 143.3–145.9) and mean DBP 85.2 mmHg (95 % CI: 84.5–85.9). In comparison, women had a mean SBP of 140.0 mmHg (95 % CI: 138.5–141.5) and mean DBP of 82.5 mmHg (95 % CI: 81.8–83.3). Mean LVM and LVMI were 236.6 g (95 % CI: 232.4–240.9) and 120.0 g/m^2^ (95 % CI: 118.1–122.1) in men and 185.0 g (95 % CI: 181.4–188.6) and 105.3 g/m^2^ (95 % CI: 103.5–107.1) in women, respectively. Detailed descriptive data can be found in Table 1.

**Fig. 1:**
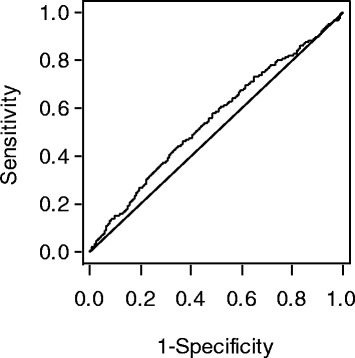
Area under the receiver operator characteristics curve (AUC) of 55.3 % (95 % CI: 52.4–58.2) of the SLI to predict LVH

For the SLI we calculated an AUC of 55.3 % (95 % CI: 52.4–58.2) to predict LVH (Fig. 1). Considering an SLI ≥ 3.5 mV as threshold to define LVH, this corresponds to a sensitivity of 5 % and a specificity of 97 %. The OR for the association of SLI with LVH was computed to be 1.59 (95 % CI: 0.74–3.42) after considering covariates indicating no statistically significant relation. The same was true for the analyses of further echocardiographic parameters (Table 2).

**Table 2:** Linear regression of echocardiographic parameters on the Sokolow-Lyon index

	Crude beta^a^ (95 % CI)	p	Adjusted beta^a,b^ (95 % CI)	p
LVMI (g/m^2^)	4.19 (1.40–6.99)	0.0033	7.02 (4.49–9.54)	<0.0001
LVIDd (cm/m^2^)	0.10 (0.08–0.12)	<0.0001	0.06 (0.04–0.08)	<0.0001
PWTd (cm)	−0.01 (−0.02–0.00)	0.1533	0.01 (0.00–0.02)	0.1078
SWTd (cm)	−0.01 (−0.02–0.00)	0.1435	0.01 (0.00–0.02)	0.0927
Dichotomized outcome for impaired echocardiographic LVH parameters [13] (odds ratios)				
LVMI	1.37 (0.67–2.79)	0.3892	1.59 (0.74–3.42)	0.233
LVIDd	1.93 (0.81–4.61)	0.1379	1.24 (0.48–3.16)	0.6572
PWTd	2 (0.48–8.29)	0.341	2.62 (0.59–11.59)	0.2041
SWTd	2.25 (0.54–9.32)	0.2633	2.66 (0.6–11.81)	0.1993

*SLI* Sokolow-Lyon index, *LVMI* left ventricular mass index, *LVIDd* left-ventricular internal diameter at end-diastole index, *PWTd* left-ventricular posterior wall thickness at end-diastole, *SWTd* septal wall thickness at end-diastole, 95 % *CI* 95 % confidence interval

^a^adjusted for sex, sport index, smoking status, NT-pro BNP, low density lipoprotein, arterial hypertension, waist-to-hip ratio, diabetes mellitus, regular intake of antiarrhythmic medication, beta blockers and digitalis glycosides

^b^Beta gives the increase of the respective echocardiographic parameter per mV increase of the SLI

Coming to linear regression models, an increase in SLI of 1 mV was associated with a significant increase in LVM of 4.2 g (95 % CI: 1.4–7.0, rounded values; *p* = 0.0033), and after covariate adjustment of 7.0 (95 % CI: 4.5–9.5). We found a significant association of SLI with echocardiographic parameters only in LVIDd-index as an increase of 0.1 cm/m^2^ per 1 mV. SWTd and PWTd showed no significant correlation (Table 2).

The Table 3 shows changes in the parameters of LVH in the different BMI classes according to the definition of the World Health Organization [16]. We found equally strong associations within different BMI classes I and II (normal and overweight), but a stronger association in class III (obese) which is supported by a significant interaction of BMI and SLI. Thus, there was evidence that the association of SLI with LVMI depends on BMI (mainly driven by class III). Considering BMI as a continuous variable, we found weak evidence (Table 3) of an interaction of BMI with the SLI in their association with the left ventricular mass index, both in the unadjusted (0.57, 95 % CI: 0.03–1.12) and covariate adjusted analysis (0.55, 95 % CI: 0.07–1.04).

**Table 3:** Linear regression of echocardiographic parameters on the Sokolow-Lyon index within considered BMI categories, and respective interaction analyses

	BMI (kg/m^2^)	Crude beta^a^ (95 % CI)	p	p (interaction)*	Adjusted beta^a,b^ (95 % CI)	p	p (interaction)*
LVMI (g/m^2^)	<25	6.34 (1.58, 11.10)	0.0092		4.79 (0.47, 9.11)	0.0041	
	25–29.9	6.09 (1.86, 10.33)	0.0049	0.0389	5.51 (1.75, 9.27)	0.001	0.0244
	≥30	9.55 (3.81, 15.30)	0.0012		9.24 (3.75, 14.73)	0.001	
LVIDd (cm/m^2^)	<25	0.05 (0.00, 0.09)	0.0384		0.03 (−0.01, 0.07)	0.1306	
	25–29.9	0.10 (0.06, 0.13)	<0.0001	0.3948	0.08 (0.05, 0.12)	<0.0001	0.1280
	≥30	0.08 (0.04, 0.12)	0.0002		0.07 (0.02, 0.11)	0.0031	
PWTd (cm)	<25	0.02 (−0.01, 0.04)	0.2255		0.01 (−0.01, 0.03)	0.4774	
	25–29.9	0.00 (−0.02, 0.02)	0.9225	0.3672	−0.01 (−0.03, 0.01)	0.5528	0.4426
	≥30	0.02 (−0.01, 0.04)	0.2604		0.02 (−0.01, 0.04)	0.2478	
SWTd (cm)	<25	0.01 (−0.01, 0.04)	0.3217		0.01 (−0.01, 0.03)	0.4411	
	25–29.9	0.01 (−0.02, 0.03)	0.624	0.6835	0.00 (−0.02, 0.02)	0.8048	0.9081
	≥30	0.01 (−0.02, 0.03)	0.4979		0.01 (−0.02, 0.03)	0.5193	

*SLI* Sokolow-Lyon index, *LVMI* left-ventricular mass index, *LVIDd* left-ventricular internal diameter at end-diastole index, *PWTd* left-ventricular posterior wall thickness at end-diastole, *SWTd* septal wall thickness at end-diastole, 95 % *CI* 95 % confidence interval

*p-value of the multiplicative interaction of the SLI with considered BMI classes

^a^adjusted for sex, sport index, smoking status, NT-pro BNP, low density lipoprotein, arterial hypertension, waist-to-hip ratio, diabetes mellitus, regular intake of antiarrhythmic medication, beta blockers and digitalis glycosides

^b^Beta gives the increase of the respective echocardiographic parameter per mV increase of the SLI

When we adjusted for age and sex only, estimates were not relevantly changed (see appendix).

## Discussion

In our study, we found a significant association of SLI with echocardiographically detected LVH. However, this association was mainly caused by an association of SLI with the LVIDd and not with the LV wall thicknesses. Although statistically significant, relations of SLI and echocardiographic parameters of LVH were weak. These relations did only depend on the BMI when obese subjects were taken into account. Furthermore, we found a very high specificity, but low sensitivity of SLI to diagnose LVH by the common threshold of 3.5 mV. Furthermore, we found a weak association of SLI with LVM, but there was no convincing relation with LVH. Thus, this finding underlines that SLI is a weak parameter to predict LVH in clinical terms and functions only weakly to assess LVM as continuous variable.

In the CARLA study collective, a high prevalence of arterial hypertension has been found [18]. In consistence, a mean LVMI of 120.0 g/m^2^ in men and 105.3 g/m^2^ in women indicates also a high burden of LVH. However, considering the former recommendations for echocardiographic quantification, this corresponds to a mild degree of LVH [13].

The low sensitivity but high specificity of SLI for LVH is consistent with published data [19, 20]. In the linear regression model, we found a significant relationship between the SLI and the LVMI (and LVIDd), although the overall relation indicated only a minor effect.

Several publications suggested a negative influence of obesity on the predictive value of the SLI [21–25]. In our study, we found significant elevations of LVMI with increasing SLI in all BMI classes, whereby the strongest association was observed in BMI class III. However, like in the whole sample, this association appeared to be mainly driven by the association of SLI and LVIDd - implicating, that SLI is primarily useful as a tool to diagnose a more eccentric type of LVH in our collective, especially in obese subjects. This might support recent findings which also implicate a higher prevalence of eccentric LVH prevailing over concentric LVH in obese patients, which might be explained by the elevated cardiac output and increased plasma volume in those patients [26].

The poor performance of the SLI has already been reported in several publications over last decades, but predominantly in hypertensive or old patient collectives [19, 20, 27–29]. In our study, we found similar results in a large general population. However, in the CARLA study collective there is a high prevalence of arterial hypertension [18].

This study has some limitations. At first, this was a retrospective analysis of prospective obtained data. All patients received a transthoracical echocardiography, which is the recommended examination for detecting LVH in recent guidelines [1]. Although it has to be stated, that in obese patients quality of echocardiographic assessments often can be poor. Furthermore, in the CARLA collective a high prevalence of arterial hypertension and also LVH (Table 1) was found and, thus, can be regarded as a selective high risk population, which is likely not representative for the general population in other regions.

## Conclusions

In conclusion, the Sokolow-Lyon index is not suitable as a diagnostic screening test to identify patients at risk for LVH, in particular in non-obese subjects without eccentric LVH. This is consistent with the recommendation of Ang and Lang from 2008 to take into consideration the ECG-findings, but only additive to a patient’s risk factors and results of 24-hour -blood pressure monitoring, to lead the right patient to echocardiography [30].

## Appendix

**Table 4:** Linear regression of echocardiographic parameters on the Sokolow-Lyon index

	Adjusted beta^a,b^ (95 % CI)	p
LVMI (g/m^2^)	5.35 (2.75–7.95)	<0.0001
LVIDd (cm/m^2^)	0.10 (0.08–0.12)	< 0.0001
PWTd (cm)	0.00 (−0.02–0.01)	0.4503
SWTd (cm)	0.00 (−0.02–0.01)	0.4804

*LVMI* left ventricular mass index, *LVIDd* left-ventricular internal diameter at end-diastole index, *PWTd* left-ventricular posterior wall thickness at end-diastole, *SWTd* septal wall thickness at end-diastole, *95 % CI* 95 % confidence interval

^a^adjusted for age and sex only

^b^Beta gives the increase of the respective echocardiographic parameter per mV increase of the SLI

**Table 5:** Linear regression of echocardiographic parameters on the Sokolow-Lyon index within considered BMI categories, and respective interaction analyses

	BMI (kg/m^2^)	Adjusted beta^a,^ ^b^ (95 % CI)	p	p (interaction)*
LVMI (g/m^2^)	< 25	5.85 (1.58–10.12)	0.0074	
	25–29.9	7.54 (3.56–11.51)	0.0002	0.0470
	≥ 30	11.65 (6.24–17.06)	<0.0001	
LVIDd (cm/m^2^)	< 25	0.04 (0.00–0.09)	0.0534	
	25–29.9	0.10 (0.06–0.13)	<0.0001	0.4554
	≥ 30	0.07 (0.03–0.11)	0.0005	
PWTd (cm)	< 25	0.01 (−0.01–0.04)	0.2290	
	25–29.9	0.01 (−0.01–0.03)	0.4673	0.4659
	≥ 30	0.02 (0–0.05)	0.0725	
SWTd (cm)	< 25	0.01 (−0.01–0.03)	0.4774	
	25–29.9	−0.01 (−0.03–0.01)	0.5528	0.8480
	≥ 30	0.02 (−0.01–0.04)	0.2478	

*LVMI* left-ventricular mass index, *LVIDd* left-ventricular internal diameter at end-diastole index, *PWTd* left-ventricular posterior wall thickness at end-diastole, *SWTd* septal wall thickness at end-diastole, *95 % CI* 95 % confidence interval

*p-value of the multiplicative interaction of the SLI with considered BMI classes

^a^adjusted for age and sex only

^b^Beta gives the increase of the respective echocardiographic parameter per mV increase of the SLI

## Abbreviations

ASE: American Society of Echocardiography
BMI: Body-mass-index
CARLA: CARdio-vascular disease, living and ageing in Halle
CI: Confidence interval
DAG: Directed acyclic graph
DPB: Diastolic blood pressure
ECG: Electrocardiogram
LVM: Left-ventricular mass
LVMI: Left-ventricular mass index
LVIDd: Left-ventricular internal diameter at end-diastole index
PWTd: Left-ventricular posterior wall thickness at end-diastole
SBP: Systolic blood pressure
SLI: Sokolow-Lyon Index
SWTd: Septal wall thickness at end-diastole
WHO: World Health Organization

## References

[1] Mancia G, Fagard R, Narkiewicz K, Redon J, Zanchetti A, Bohm M (2013). 2013 ESH/ESC guidelines for the management of arterial hypertension: the Task Force for the Management of Arterial Hypertension of the European Society of Hypertension (ESH) and of the European Society of Cardiology (ESC). Eur Heart J.

[2] Wolf-Maier K, Cooper RS, Banegas JR, Giampaoli S, Hense HW, Joffres M (2003). Hypertension prevalence and blood pressure levels in 6 European countries, Canada, and the United States. JAMA.

[3] Meisinger C, Heier M, Volzke H, Lowel H, Mitusch R, Hense HW (2006). Regional disparities of hypertension prevalence and management within Germany. J Hypertens.

[4] Koren MJ, Devereux RB, Casale PN, Savage DD, Laragh JH (1991). Relation of left ventricular mass and geometry to morbidity and mortality in uncomplicated essential hypertension. Ann Intern Med.

[5] Schillaci G, Battista F, Pucci G (2012). A review of the role of electrocardiography in the diagnosis of left ventricular hypertrophy in hypertension. J Electrocardiol.

[6] Sokolow M, Lyon TP (1949). The ventricular complex in left ventricular hypertrophy as obtained by unipolar precordial and limb leads. Am Heart J.

[7] Greiser KH, Kluttig A, Schumann B, Kors JA, Swenne CA, Kuss O (2005). Cardiovascular disease, risk factors and heart rate variability in the elderly general population: design and objectives of the CARdiovascular disease, Living and Ageing in Halle (CARLA) Study. BMC Cardiovasc Disord.

[8] Haerting J, Kluttig A, Greiser KH, Nuding S, Werdan K (2012). A cohort study investigating risk factors for cardiovascular disease in an urban elderly East-German population (CARLA study). Bundesgesundheitsblatt Gesundheitsforschung Gesundheitsschutz.

[9] van Bemmel JH, Kors JA, van Herpen G (1990). Methodology of the modular ECG analysis system MEANS. Methods Inf Med.

[10] Kors JA, van Herpen G, Wu J, Zhang Z, Prineas RJ, van Bemmel JH (1996). Validation of a new computer program for Minnesota coding. J Electrocardiol.

[11] De Bruyne MC, Hoes AW, Kors JA, Hofman A, Van Bemmel JH, Grobbee DE (1999). Prolonged QT interval predicts cardiac and all-cause mortality in the elderly The Rotterdam Study. Eur Heart J.

[12] Lang RM, Bierig M, Deverux RB, Flachskampf FA, Foster E, Pellikka PA (2005). Recommendations for chamber quantification: a report from the American Society of Echocardiography’s Guidelines and Standards Committee and the Chamber Quantification Writing Group, developed in conjunction with the European Association of Echocardiography, a branch of the European Society of Cardiology. J Am Soc Echocardiogr.

[13] Lang RM, Bierig M, Deverux RB, Flachskampf FA, Foster E, Pellikka PA (2006). Recommendations for chamber quantification. Eur J Echocardiogr.

[14] Baecke JA, Burema J, Frijters JE (1982). A short questionnaire for the measurement of habitual physical activity in epidemiological studies. Am J Clin Nutr.

[15] Textor J, Hardt J, Knüppel S (2011). DAGitty: a graphical tool for analyzing causal diagrams. Epidemiology.

[16] World Health Organization (1995). Physical status: the use and interpretation of anthropometry. Report of a WHO Expert Committee. World Health Organ Tech Rep Ser.

[17] Tiller D, Russ M, Greiser KH, Nuding S, Kluttig A, Ebelt H (2013). Prevalence of symptomatic heart failure with reduced and with normal ejection fraction in an elderly general population - the CARLA study. PLoS One.

[18] Kluttig A, Schumann B, Swenne CA, Kors JA, Kuss O, Schmidt H (2010). Association of health behaviour with heart rate variability: a population-based study. BMC Cardiovasc Disord.

[19] Somaratne JB, Whalley GA, Poppe KK, ter Bals MM, Wadams G, Pearls A (2011). Screening for left ventricular hypertrophy in patients with type 2 diabetes mellitus in the community. Cardiovasc Diabetol.

[20] Cuspidi C, Facchetti R, Bombelli M, Sala C, Grassi G, Mancia G (2014). Accuracy and prognostic significance of electrocardiographic markers of left ventricular hypertrophy in a general population: findings from the Pressioni Arteriose Monitorate E Loro Associazioni population. J Hypertens.

[21] Abergel E, Tase M, Menard J, Chatellier G (1996). Influence of obesity on the diagnostic value of electrocardiographic criteria for detecting left ventricular hypertrophy. Am J Cardiol.

[22] Levy D, Labib SB, Anderson KM, Christiansen JC, Kannel WB, Castelli WP (1990). Determinants of sensitivity and specificity of electrocardiographic criteria for left ventricular hypertrophy. Circulation.

[23] Okin PM, Jern S, Devereux RB, Kjeldsen SE, Dahlof B (2000). Effect of Obesity on Electrocardiographic Left Ventricular Hypertrophy in Hypertensive Patients : The Losartan Intervention For Endpoint (LIFE) Reduction in Hypertension Study. Hypertension.

[24] Okin PM, Devereux RB, Jern S, Kjeldsen SE, Julius S, Dahlof B (2000). Baseline characteristics in relation to electrocardiographic left ventricular hypertrophy in hypertensive patients The Losartan Intervention For Endpoint Reduction (LIFE) in hypertension study. Hypertension.

[25] da Costa W, Perez Riera AR, de Assis CF, Nogueira Bombig MT, de Paola AAV, Camargo Carvalho AC (2008). Correlation of electrocardiographic left ventricular hypertrophy criteria with left ventricular mass by echocardiogram in obese hypertensive patients. J Electrocardiol.

[26] Cuspidi C, Rescaldani M, Sala C, Grassi G (2014). Left-ventricular hypertrophy and obesity: a systematic review and meta-analysis of echocardiographic studies. J Hypertens.

[27] Denarie N, Linhart A, Levenson J, Simon A (1998). Utility of electrocardiogram for predicting increased left ventricular mass in asymptomatic men at risk for cardiovascular disease. Am J Hypertens.

[28] Pewsner D, Juni P, Egger M, Battaglia M, Sundstrom J, Bachmann LM (2007). Accuracy of electrocardiography in diagnosis of left ventricular hypertrophy in arterial hypertension: systematic review. BMJ.

[29] Gosse P, Jan E, Coulon P, Cremer A, Papaioannou G, Yeim S (2012). ECG detection of left ventricular hypertrophy: the simpler, the better?. J Hypertens.

[30] Ang D, Lang C (2008). The prognostic value of the ECG in hypertension: where are we now?. J Hum Hypertens.

